# The Cerebral Palsy Link Worker (CP LINK) study: Protocol for a feasibility study with integrated process evaluation

**DOI:** 10.1371/journal.pone.0343206

**Published:** 2026-02-23

**Authors:** Kimberley J. Smith, Erica Ranzato, Miriam Creeger, Valerie L. Stevenson, Karen Lowton, Cherry Kilbride, Meriel Norris, Christina Victor, Thelonious McCarthy Budzynski, Emma Livingstone

**Affiliations:** 1 School of Psychology, University of Surrey, Guildford, Surrey, United Kingdom; 2 UP – The Adult Cerebral Palsy Movement, London, United Kingdom; 3 National Hospital for Neurology and Neurosurgery, UCLH, London, United Kingdom; 4 University of Sussex, Brighton, United Kingdom; 5 Brunel, University of London, Uxbridge, Middlesex, United Kingdom; PLOS: Public Library of Science, UNITED KINGDOM OF GREAT BRITAIN AND NORTHERN IRELAND

## Abstract

**Background:**

Cerebral palsy (CP) is the most common lifelong physical disability in the UK and is linked to multiple social, psychological and health inequalities which are often amplified as this group ages. Most middle-aged and older people with CP are supported in their community by General Practitioners (GPs) and complain of disjointed and non-specialised care from people who have a poor understanding of their complex ageing needs. This points to the need for specialised holistic care for this group. Therefore, there is a pressing need to develop ways to support people ageing with CP within their communities.

**Project aim:**

To evaluate a co-developed specialised CP link worker role to support adults aged 40 and older ageing with CP in North Central London over 1 year.

**Methods:**

We will undertake an evaluation of the link worker role by gathering data at baseline, 1-week and 3-months from participants who use the service. Feasibility outcomes will include recruitment and retention rates, acceptability of the intervention, completeness and quality of outcome data, and the practicality of collecting and analysing participant-reported outcome measures. Process evaluation outcomes will include insights into how the intervention was delivered and received, fidelity to the intervention model, contextual factors that influenced implementation, potential sustainability, and stakeholder experiences. This project has been co-developed with the charity UP – The Adult Cerebral Palsy Movement, and we will work alongside a lived experience advisory group and stakeholder advisory group throughout the project.

## Introduction

There are 130,000 adults ageing with cerebral palsy (CP) in the UK, and existing research suggests this group experiences lifelong issues with multiple social determinants of health including decreased social participation, lower employment, social exclusion, discrimination, and a lack of access to specialised health services in adulthood [1–5]. A lifetime of inequality increases the risk of developing physical, social, and mental health issues as people with CP age.

Adults ageing with CP are more likely to develop serious physical age-related issues at much earlier ages than we see in the general population. In their late 30s and early 40s it is common for people with CP to require joint replacements, experience multiple falls and need to use mobility aids [6,7]. Alongside this we observe people with CP being more likely to develop a range of age-related health issues much earlier such as heart and respiratory diseases, diabetes, arthritis, and dementia alongside the worsening of lifelong symptoms such as pain and fatigue [8–11]. These physical health issues can exacerbate social and mental health difficulties for older adults with CP. Issues such as loneliness [12,13] social isolation and social exclusion [14] and mental illness [15] are all commonly reported in this ageing population.

These complex physical, social, and mental health issues point to the need for specialised support. In childhood people with CP are often supported by specialist teams, however, once people reach adulthood they are often discharged from these services and transitioned to non-specialised care; something that many people with CP struggle with [16]. At this point it is a primary care clinician (referred to as a General Practitioner (GP)  in the UK) rather than a specialist clinician who becomes the main point of contact and healthcare support for adults with CP in the UK [3]. Although research has focused on the ways to support adults making the transition from specialised childhood care to non-specialised adult care [16,17], there remains little understanding of the meaningful ways we can support older adults with CP who are relying on non-specialist support within their community. As the GP is the main healthcare contact for adults with CP, there is a need for us to look to innovative ways to aid primary care in supporting this ageing population within the community.

We are framing our project through a lens of healthy ageing, a process that allows people to maintain their wellbeing in older age through maintenance of functional ability [18]. To enjoy a good older age, it is important that people have the lifelong support needed to age healthily – physically, socially, and mentally. However, the health inequalities experienced by people with CP suggest we need to develop better ways to support this group to age well. Social prescribing is one way we could support people with CP within their community to promote their health and wellbeing and reduce health inequalities.

Social prescribing aids emotional, social and practical needs [19] and includes being referred/introduced by a link worker to specific/organised activities such as befriending, volunteering opportunities, supported education, creative arts, physical activity, and support with employment or legal advice. There are ongoing studies that are piloting and testing the feasibility of specialised link worker roles for populations such as those with depression, cancer and pain [20–22]. Therefore, there is a unique and timely opportunity for us to develop a link worker service specifically targeted at the needs of adults ageing with CP.

While there are some specialist centres that support adults with CP in the UK, these are often in tertiary centres and people cannot rely on these services for day-to-day support for their wellbeing. There is a need for us also to consider how we can implement specialised support that will be more readily accessible to people ageing with CP within their own community.

As such, the overarching aim of this project is to assess the feasibility and acceptability of delivering a link worker intervention for middle-aged and older adults with CP living in North Central London, including the feasibility of collecting outcome data. It also aims to conduct a process evaluation to explore how the intervention is delivered, received, and shaped by contextual factors. We have the following specific objectives:

1.) Establish the reach, retention, dose, fidelity, acceptability, potential sustainability, contextual influences, and any unintended consequences of the intervention.
2.) Document the type and range of community-based groups and activities prescribed by the CP link worker to participants.
3.) Identify and explore the experiences and engagement of participants and key stakeholders involved in the delivery of the intervention.
4.) Evaluate the methodological and procedural aspects of the intervention, including recruitment strategies, consent procedures, and data collection methods.
5.) Assess the suitability of the selected outcome measures for evaluating the impact of the intervention.

## Methods

### Co-design of project

This project has been co-developed with the charity UP – The Adult Cerebral Palsy Movement (UP) [23]. UP are a highly research active charity who are keen to develop research that will have direct impact on the lives of adults with CP. UP have highlighted the disparate and lack of joined up care for adults with CP, particularly those older adults with CP whose health needs have worsened. They state that most older adults with CP are supported by GPs who do not understand their unique and heterogeneous ageing needs. They also emphasise that there are potentially many resources that could be available within the community to support older adults with CP, but that these need to be mapped, and there needs to be a way to signpost older adults with CP to these services. Finally, they state that adults ageing with CP and healthcare providers are not always aware of how CP might impact them as they get older, and that a link worker could have an important role in educating both adults with CP, GP’s and their broader communities about the impact of ageing. Thus, we opted to pilot a specialised link worker role over other potential interventions as when we have spoken to adults with CP, they are keen that they receive person-centred support for their ageing needs.

### Patient and Public Involvement (PPI)

Alongside working with UP as project partners we are also working closely with two advisory groups. Our lived experience advisory group consists of 6 adults with CP who are advising on the implementation of the project. The group will meet regularly over the course of the project and advise on key aspects of the project such as interview and questionnaire development, study recruitment, aiding the interpretation of study data and helping develop a project dissemination plan. Our stakeholder advisory group consists of 4 healthcare professionals working with adults with CP and people who work in social prescribing. This group supports the team with ideas for recruitment, development of training resources for the link worker, dissemination and advising on post-project implementation. Alongside these groups we have involved additional adults with CP (n = 5), link workers (n = 4) and health and social care professionals (n = 5) in co-development workshops over Summer 2025 to develop training for the link worker and give advice on project implementation.

### Enhancing accessibility

To enhance accessibility of the study we will produce easy-read versions of all participant documents. We will also produce large font versions of materials. The need for alternative language materials will also be assessed and met throughout the project by working with professional translation services (including British Sign Language). Participants will be offered alternative options for data collection including online, by phone, hard copy or in person.

### Intervention study

#### Design.

This study is a feasibility study with integrated process evaluation which will collect both quantitative and qualitative data.

#### Participants and recruitment.

Two pools of participants will be recruited: – (a) adults with CP who take part in the intervention and (b) key stakeholders and supporters who have been involved in the development or delivery of the intervention.

For the intervention sample we will recruit adults with CP aged 40 and older living in North Central London (NCL, i.e., the boroughs of Barnet, Camden, Enfield, Haringey, and Islington) who have capacity to consent, or their carer/ supporter responding on their behalf. We aim to recruit a minimum of 31 adults with CP to the intervention. Sample sizes between 12 and 50 have been recommended for pilot and feasibility studies [24,25]. A power analysis for a within-groups repeated measures analysis of variance (ANOVA) with 0.8 power, a medium effect size and 3 measures (baseline, 1 week after the start of the intervention, 3 months after the start of the intervention) yielded a size of 20. With a drop-out rate of 30% we will need a minimum of 31 people. Participants will need to live in the North Central London (NCL) Integrated Care System (ICS) area. We will identify potential participants through participant identification centres (NHS trusts) that are accessed by people with CP. Recruitment will also be conducted by the research team via social media channels and through distribution of leaflets in community-based organizations operating in NCL.

For the key stakeholders and supporters sample we will invite people involved in the development or delivery of the intervention to take part in semi-structured interviews to share their feedback on the CP Link Worker intervention. Individuals who have held one of the following roles will be eligible: link worker, community-based service providers to whom participants were referred as part of the social prescriptions, participant’s carer/ supporter, or UP founder involved in the training and supervision of the link worker.

This study will recruit participants into the intervention from 21/01/2026 until 31/10/2026. Data collection will be completed by January 2027 and data analysis will be completed by April 2027.

#### Link worker intervention setting.

The NCL ICS supports a diverse population of 1.5 million people across five London boroughs. This site supports many people within a geographically constrained area increasing the feasibility of us identifying adults with CP and mapping community resources. The effectiveness of the link worker in an area of this size will be extremely informative for other ICSs. Potential community resources and needs have been identified through a literature search, consultation with advisory groups and examining results from a qualitative study where we interviewed 22 adults aged 40 and older living in London about their experiences of ageing and use of community resources [26]. We have also mapped community resources in NCL and online resources that could be well placed to meet these different non-medical needs (e.g., community resources that could support someone experiencing pain). These resources will be shared with the link worker who will continue to update and map additional resources during the intervention.

#### Link worker intervention.

**Fig 1:**
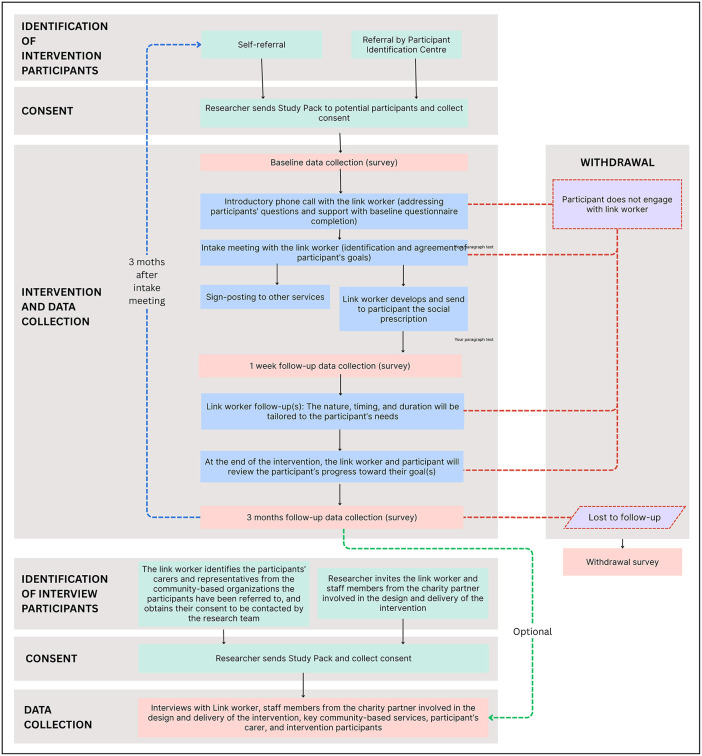
Flowchart of the link worker intervention, including recruitment, data collection, and intervention phases.

The intervention will involve a specialised link worker role based in NCL for a period of 12 months. This link worker will receive bespoke training on living and ageing with CP plus any additional training needs identified by themselves and UP (e.g., motivational interviewing). Following this they will work in the NCL ICS. The link worker will work with adults with CP to determine their individual non-medical needs and then link them into community resources that could support them to improve their health and wellbeing. The process of the link worker intervention is provided in Fig 1.

Once informed consent is provided to the postdoctoral researcher (ER), the link worker will contact the eligible participant to arrange the introductory phone call and will send them either the link or the paper version of the baseline questionnaire, which must be completed before the intake meeting. During the introductory phone call the link worker will answer any questions the participant may have in relation to their role and will check whether the baseline questionnaire has been completed. If the survey has not been completed, the link worker will provide assistance before moving forward. If the participant has questions about the study itself, the researcher will be available to answer these and, if needed, arrange a separate meeting with the participant.

After the introductory phone call, the link worker will schedule the intake meeting. This can take place in person, over the phone, or via computer (video call), depending on the participant’s preference. During the intake meeting, the link worker will work with the participant to identify their non-medical needs and main priority areas, and to establish an agreed set of priority goals to be achieved within the intervention. Goals will be tailored to each participant’s needs and should follow the SMART principle – Specific, Measurable, Attainable, Realistic and Timely – with the overall aim of supporting the participant’s wellbeing. To help focus the discussion and ensure areas of needs are prioritised, participants will be asked to identify their top two concerns using a structured tool, based on Goal Attainment Scaling [27] and adapted from existing practice. This tool provides a guide to support the identification and prioritisation of needs, while also allowing flexibility to address other areas the participant wishes to raise. The process of identifying and prioritizing non-medical needs and goals will remain participant led. The intake meeting will last approximately 1 hour.

Based on the intake meeting, the participant’s identified non-medical needs, and the agreed goals, the link worker will develop a social prescription plan detailing recommended community-based activities and services tailored to the participant’s needs. The social prescription will be shared with the participant electronically or in print, based on their preference. The nature and type of community-based resources recommended by the link worker may vary depending on availability at the time of the intervention. The link worker will follow up with the participant after the intake meeting to check on their progress and access to the referred services, to offer additional support if needed, and to help connect them to the community-based services. Due to the person-centred nature of the link worker services, it is difficult to define a standardised duration for the intervention and follow-up(s), as the length and intensity of support are shaped by each participant’s needs and the complexity of their circumstances [28]. As a result, the timing and number of follow-up contacts will be flexible and tailored to each participant’s needs and level of complexity. Some participants may require brief, short-term contacts, while others may need ongoing or intermittent support. The schedule and format of such follow-up contacts will be agreed between the participant and the link worker.

Link worker support will be available for up to three months, although it may finish earlier if the participant and the link worker agree that the intervention has been completed. At the end of the intervention, the link worker will schedule a meeting with the participant to review the progress made and the extent to which their goals have been achieved. This can take place in person, over the phone, or via computer (video call), depending on the participant’s preference.

If a participant presents with several unrelated non-medical needs, it may be necessary to conduct more than one intake meeting, each resulting in a separate cycle of goal setting and corresponding social prescription. The determination of whether needs are sufficiently unrelated to warrant separate intervention cycles will be made at the discretion of the link worker, based on their professional judgement. To ensure that each cycle can be evaluated separately, each intake meeting (and corresponding social prescription) should occur at least three months after the previous one.

If a participant presents with clinical needs outside the scope of social prescribing, the link worker will escalate the case to the designated supervisor or appropriate staff at UP, who oversee the social prescribing service.

Participants will be encouraged to bring along a supporter or carer should this help them feel more comfortable during any part of the intervention.

#### Link worker role and responsibilities.

In addition to supporting participants, the link worker will be responsible for aspects of data collection, including recording meeting notes (field notes) and assisting with questionnaire administration. The questionnaires and field notes are described in more detail below.

The link worker will also be responsible for maintaining and updating a database of community-based activities and services in NCL, and for connecting with these services. The database includes two components: (1) the list of community resources described above and (2) a selection of directories relevant to social prescribing practice. Throughout the one-year intervention, the link worker will update and add new resources in response to participants’ emerging needs as well as changes in service provision within NCL and will build relationships with local service providers to ensure referral options are relevant and accessible to participants.

Finally, the link worker will contribute to recruitment activities. As part of their role, the link worker will identify and engage with local healthcare and community services within NCL to promote the link worker programme and provide information about the referral process. Moreover, they will help identify and invite community-based service providers and participants’ carers/ supporters to take part in interviews as part of the intervention evaluation at the end of the intervention. The interviews are described in more detail below.

#### Outcome measures.

This study will use a mixed-methods approach, combining quantitative and qualitative data collected through different sources.

Based on previous process evaluations of social prescribing interventions [29,30] and education programmes [31], the process evaluation of the CP link worker intervention will examine reach, dose, fidelity, acceptability, sustainability, and unintended consequences as defined below.

Reach is defined as the extent to which the intended population engages with the intervention. It will be assessed using quantitative data on uptake (e.g., agreement to be referred to the link worker) and engagement (e.g., continued participation in link worker appointments and use of referred community services). Participants’ characteristics will also be analysed to explore potential patterns in engagement and disengagement. To support this, the following data will be collected:

- Number of participants invited by the participant identification centres (NHS trusts) to join the link worker programme and who self-referred
- Number of participants meeting eligibility criteria
- Number of participants enrolled in the programme
- Referral records (date, referral source, reasons for referral)
- Sociodemographic and clinical characteristics of participants
- Use of health and social care services
- Retention rates.

Dose is defined as the number, format (in person, on-line, phone), type (introductory, intake, follow up), and duration of meetings between the participant and the link worker. It will be recorded and assessed using qualitative and quantitative data on the link worker activity collected through the link worker field notes.

Fidelity is defined as the extent to which the intervention is delivered as intended. It will be assessed using both quantitative and qualitative data on the link worker activity, primarily collected through link worker field notes. This will include whether the introductory phone call and the intake meeting were completed, whether participant non-medical needs were identified and goals were set, the number and types of community resources recommended, whether and when the community resources were accessed by participants including reasons for non-access, the number and modality of follow-up contacts made, and whether participants’ goal achievement was reviewed. Participant access to community resources, including timing, waiting periods, and frequency of use, will be triangulated with data from the 3-months follow-up questionnaire to strengthen the assessment of fidelity.

Acceptability is defined as the extent to which the intervention is perceived as appropriate and satisfactory by participants and stakeholders, based on their experiences of its content, delivery, and perceived value. It will be assessed through a 3-month follow-up questionnaire with participants, and they will also be invited to take part in a voluntary interview. In addition, participant dropout and non-engagement rates will be recorded, along with reasons for withdrawal or disengagement, when possible. Semi-structured interviews will be conducted with the link worker, community-based service providers, participant’s carer/ supporter, and the founders of UP to explore perceived acceptability of the intervention.

Potential sustainability is defined as the extent to which the CP link worker intervention can be maintained or embedded within routine practice over time. It will be assessed through interviews with the link worker, community-based service providers, and the founders of UP, exploring perceptions of feasibility for long-term implementation, resource requirements, organisational capacity, and alignment with existing systems.

Unintended events or consequences of the CP link worker intervention will be recorded and explored through the link worker field notes and through the interviews with participants, their carers/ supporters, link worker, community-based service providers, and the founders of UP.

#### Data collection.

The number of people invited to take part in the intervention, those who self-refer, the number of participants enrolled in the programme, and retention rates will be recorded by the researcher throughout the intervention period.

Data will be collected from participants through survey at three time points – baseline, 1-week, and 3-month follow-up – and through a voluntary-based interview at the end of the intervention. Participants will be able to complete the survey either online or on paper. Participants will be asked to complete the survey within one week of receiving it. Participants who require additional support may choose to complete the survey with assistance from their carer/ supporter, the link worker, or request a paper version of the questionnaire by post. The questions asked at baseline, 1-week and 3-months are outlined in Table 1.

**Table 1:** Data collected from intervention participants.

Domain	Measure	Baseline questionnaire	Intake meeting	1-week follow up questionnaire	3-month follow up questionnaire
Sociodemographic and clinical information	Study-specific items	✓	–	–	–
Use of health and social care services	Use of health and social care services (Understanding Society survey)	✓	–	–	✓
Achievement of participant goals	Goal Attainment Scaling – light model (GAS- Light) [27]	–	✓	–	✓
Psychological wellbeing	World Health Organization-Five Well-Being Index (WHO-5) [32]	✓	–	–	✓
Health related quality of life	EuroQol 5-Dimension 5-Level (EQ-5D-5L) [33]	✓	–	–	✓
Loneliness	UCLA-3 item Loneliness Scale (UCLA-3item) and a direct question about how often the respondent feels lonely [34]	✓	–	✓	✓
Fatigue	One-Item Fatigue Screen (OIFS) [35]	✓	–	✓	✓
Pain	Single item from EQ-5D-5L [33]	✓	–	–	✓
Social network	Lubben social network scale [36]	✓	–	–	✓
Social group membership and sense of local community	Selected items adapted from [37]	✓	–	✓	✓
Empowerment	Adapted version of the Diabetes Empowerment Scale – Short Form (DES-SF) [38] Individual community-related empowerment scale: Dimension 2 (intention) and 3 (participation) [39]	✓	–	✓	✓
Feedback on the experience with the link worker	NHS Patient Social Prescribing Link Worker Feedback [40]	–	–	✓	✓ (*)
Feedback on social prescription	Study-specific items	–	–	–	✓ (*)
Feedback on the overall intervention	Study-specific items	–	–	–	✓ (*)

Footnote for Table 1: (*) Component also explored in the optional interview at the end of the intervention. Different questionnaires are administered at 1-week and 3-months due to the fact that the 1-week follow-up collects data on outcomes related to the link worker and those measures that require the participant to think about the outcome for a week or less (those questionnaires that require the participant to think about the outcome for more than a week are asked about at 3-months only). The need for different questionnaires at 1-week and 3-months is also due to the fact that at 1-week the participant will be reflecting primarily on contact with the link worker and that at 3-months the participant will be reflecting on contact with the link worker and social prescription (as PPI feedback has indicated that it can take a few weeks for participants to access their social prescriptions).

Throughout the intervention period the link worker will complete field notes – brief written records made during or after interactions with participants or while delivering the intervention. These notes will capture the date, modality, timing, and duration of each interaction, as well as observations, reflections, challenges, and contextual details relevant to the delivery of the intervention. Field notes will be reviewed periodically by the research team to ensure the quality of data collection and to assess adherence to the delivery model, and any adaptations or deviations will be documented along with the rationale for these changes. In addition to the field notes, the link worker will complete an intake form and a social prescription plan for each participant.

After completing the 3-month follow up questionnaire, all participants will be invited to take part in an interview with the research team to discuss their expectations of the intervention, their experiences with the link worker, and the activities to which they were referred. The interview will also explore their views on what worked well and what could be improved. Participation is voluntary, and refusal will not affect involvement in the study. The interview will be conducted either online, over the phone, or in-person depending on the preference of the interviewee and will last around 60 minutes. To help reduce potential anxiety, the list of interview questions will be shared with interviewees one day before the interview. The accompanying message will clarify that no preparation is required for the interview unless the interviewee chooses to do so. Interviewees will also be able to contact the research team up to one week after the interview to share any additional thoughts or reflections.

Withdrawal from the intervention will be defined as either an explicit decision communicated by the participant (or their carer/ supporter) to the research team to discontinue their involvement (active withdrawal) or non-engagement despite repeated follow-up attempts (passive withdrawal). Passive withdrawal will be defined as non-engagement after three unsuccessful follow-up attempts by the link worker within one month, using a combination of methods (phone, email, and post). No further contact will be made by the link worker after this point. Withdrawal may occur at one of three stages: prior to the introductory phone call with the link worker, after receiving the social prescription, or during the 3-months follow-up period for data collection. In the event of participant withdrawal, the research team will invite participants to complete a brief withdrawal questionnaire online or on paper to capture the reason for disengagement and gather feedback on the intervention. In case of non-engagement, the reason for withdrawal will be recorded as unknown.

At the end of the intervention, semi-structured interviews will be conducted with the link worker, community-based service providers, participants’ carers/ supporter, and the founders of UP involved in the training and supervision of the link worker. Interviews will be conducted online, over the phone, or in-person depending on the preference of the interviewee. The same interview procedures used with intervention participants will be used with stakeholders, including advance sharing of interview questions and a one-week window for post-interview reflections.

The development of the questionnaires and the interview topic guides has been informed by previous evaluation studies of social prescribing interventions [29,37,41,42], as well as by discussions with the study’s advisory groups. All questionnaires have been piloted with members of the lived experience advisory group,

Copies of the questionnaires and of the interview topic guides are included on the Open Science Framework [43].

#### Data analysis.

Normally distributed variables will be summarised by means and standard deviations, skewed continuous variables by medians and interquartile ranges and categorical variables by frequencies and percentages.

All interviews will be transcribed verbatim, and all participant data will be pseudo-anonymised by assignment of a unique study ID. Qualitative data will be analysed using thematic analysis [44].

### Ethical considerations

We have obtained ethical approval from the Health Research Authority and Health and Care Research Wales North-West Greater Manchester research ethics committee (25/NW/0364). NHS research and development approvals will be sought from all participant identification centres prior to recruitment commencing from that site.

As a team we also need to consider whether additional safeguarding procedures need to be put in place during the study. We have created protocols to assess capacity in participants with learning disabilities, so that we are able to only include those people in the study who are able to provide informed consent.

The link worker will be expected to work within the ethical, working practices and health and safety of different recruitment sites. This will be discussed in advance with each site to ensure that their protocols are adhered to.

Additional ethical concerns can also be raised around co-design. We need to be mindful of power dynamics and ensure that participation is equitable and democratic. We also need to consider intellectual property for any co-designed outputs. To acknowledge their participation we will offer to name any contributor on project outputs.

### Data management

The team will adhere to the principles of the GDPR and University of Surrey policies that are aligned to comply with the Data Protection Act 2018. All data covered by the GDPR will be password protected and kept in a secure University file space. Only the link worker (TMB), staff overseeing the link worker intervention (EL, MC), principal investigator (KS) and post-doctoral researcher (ER) will have access to any non-anonymized data.

All participants will be given a unique pseudonym and participant ID which will be used to identify them on any study documentation. If any participant provides personally identifiable data (e.g., names) for other people/ key places in interviews, this information will be changed in all transcripts. We will also not use any quotes from participants which could make them identifiable. Any identifiable information will be stored separately to anonymized data and records of informed consent.

As per the requirement of our funder we aim to share anonymized data from those participants who consent to this at the end of the project. Prior to this any information that could render a person identifiable will be redacted by the project team. Where participants have not consented to wider sharing, we will remove their data prior to curating and archiving that specific form of data.

### Dissemination and data sharing

Data arising from the study will be the intellectual property of the University of Surrey. Findings from this study will be disseminated through an academic paper for publication in a peer-reviewed journal, at least one presentation at a scientific conference. We will also produce simplified study summaries for members of the public. Findings from this study will be promoted via the project partners (UP) and interested participants will be given the opportunity to contact the postdoctoral researcher to obtain a copy of these.

We will also share anonymised data for those participants who consent to have this data shared via the University of Surrey Open Access platform (ESPLORO).

Finally, we will create a toolkit for link workers with information about supporting people ageing with CP.

## Discussion

While this project is not a randomised controlled trial and is not able to formally determine the effectiveness of this intervention, it will allow us to gather initial data on whether a specialised link worker is a feasible and promising approach to support people ageing with CP. Alongside this, the research allows us to address broader inequalities faced by adults with CP in terms of specialised research and care. The number of adults ageing with CP is equivalent to those ageing with multiple sclerosis or Parkinson’s disease, however adults with CP have significantly fewer specialised healthcare pathways, resources, and research than these other conditions. There is also an inequality with the CP research and care community as most research into improving care and wellbeing focuses on younger people with CP around the time of transition to adult services. There is a lot less research that focuses specifically on improving the care provided to older adults with CP. Conducting this project allows us to ‘level up’ the support offered to a group who have significantly fewer healthcare resources allocated to them.
